# Predicting the Recurrence of Gastric Cancer Using the Textural Features of Perigastric Adipose Tissue on [^18^F]FDG PET/CT

**DOI:** 10.3390/ijms231911985

**Published:** 2022-10-09

**Authors:** Hyein Ahn, Geum Jong Song, Si-Hyong Jang, Myoung Won Son, Hyun Ju Lee, Moon-Soo Lee, Ji-Hye Lee, Mee-Hye Oh, Geum Cheol Jeong, Jong Hyuk Yun, Sang Mi Lee, Jeong Won Lee

**Affiliations:** 1 Department of Pathology, Soonchunhyang University Cheonan Hospital, 31 Suncheonhyang 6-gil, Dongnam-gu, Cheonan 31151, Korea; 2 Department of Surgery, Soonchunhyang University Cheonan Hospital, 31 Suncheonhyang 6-gil, Dongnam-gu, Cheonan 31151, Korea; 3 Department of Nuclear Medicine, Soonchunhyang University Cheonan Hospital, 31 Suncheonhyang 6-gil, Dongnam-gu, Cheonan 31151, Korea; 4 Department of Nuclear Medicine, College of Medicine, Catholic Kwandong University, International St. Mary’s Hospital, 25 Simgok-ro 100-gil, Seo-gu, Incheon 22711, Korea

**Keywords:** adipose tissue, F-18 fluorodeoxyglucose, positron emission tomography, stomach neoplasm, textural feature

## Abstract

This study aimed to assess the relationship between the histopathological and textural features of perigastric adipose tissue (AT) on 2-deoxy-2-[^18^F]fluoro-D-glucose ([^18^F]FDG) positron emission tomography/computed tomography (PET/CT) and to evaluate the prognostic significance of perigastric AT textural features in predicting recurrence-free survival (RFS) in patients with gastric cancer. Sixty-nine patients with gastric cancer who underwent staging [^18^F]FDG PET/CT and subsequent curative surgery were retrospectively reviewed. Textural features of perigastric AT were extracted from PET images. On histopathological analysis, CD4, CD8, and CD163 cell infiltration and matrix metalloproteinase-11 and interleukin-6 (IL-6) expression in perigastric AT were graded. The degree of CD163 cell infiltration in perigastric AT was significantly correlated with the mean standardized uptake value (SUV), SUV histogram entropy, grey-level co-occurrence matrix (GLCM) energy, and GLCM entropy of perigastric AT. The degree of IL-6 expression in the perigastric AT was significantly correlated with the mean and median SUVs of perigastric AT. In multivariate survival analysis, GLCM entropy, GLCM dissimilarity, and GLCM homogeneity of perigastric AT were significant predictors of RFS. The textural features of perigastric AT on [^18^F]FDG PET/CT significantly correlated with inflammatory response in perigastric AT and were significant prognostic factors for predicting RFS in patients with gastric cancer.

## 1. Introduction

Although the incidence and mortality rates of gastric cancer have been reported to decline over the past decades, gastric cancer remains to be the fifth most prevalent cancer and one of the leading causes of cancer-related death globally [1]. In gastric cancer patients without distant metastasis, curative gastric resection and regional lymph node dissection are the recommended standard treatments [2]. However, even in gastric cancer patients who have undergone curative surgical resection, a significant number experienced cancer recurrence after surgery, showing a 3-year disease-free survival rates of only 61.2–66.1% [3,4]. Therefore, a number of studies have tried to identify clinicopathological and imaging biomarkers of gastric cancer to predict the risk of recurrence [5]. Recently, several studies have focused on the role of interactions between cancer cells and peritumoral adipose tissue (AT) cells in cancer progression [6,7]. Through the interactions, adipocytes at the cancer invasion front transforme into cancer-associated adipocytes, which exhibit different morphological and functional features compared with normal adipocytes, and these cancer-associated adipocytes induce inflammatory responses in the AT and promote cancer cell proliferation and progression [6,7,8]. Because gastric cancer cells grow in an adipocyte-predominant environment, previous studies have demonstrated profound interactions between gastric cancer cells and peritumoral AT [6]. In a histopathological analysis of surgical specimens, AT adjacent to gastric cancer showed different features from AT that was distant from the tumor [9]. Furthermore, adipocytes in visceral AT were found to enhance invasiveness and metastasis and modulate the chemosensitivity of gastric cancer cells [10,11].

2-Deoxy-2-[^18^F]fluoro-D-glucose ([^18^F]FDG) positron emission tomography/computed tomography (PET/CT) is an imaging modality that has shown clinical values for staging, detecting recurrence and predicting prognosis in patients with gastric cancer [5,12,13,14]. In addition to detecting malignant lesions, since [^18^F]FDG PET/CT can be used to evaluate glucose metabolism in an organ, several studies have demonstrated the clinical use of [^18^F]FDG PET/CT in estimating the host response to cancers [15,16]. In previous studies, PET imaging parameters of AT were significantly associated with the presence of lymph node metastases and clinical outcomes in diverse cancers, suggesting that PET imaging parameters of AT could be used as surrogate markers of inflammatory response in AT [16,17,18]. Since gastric cancer cells also have a substantial interrelationship with peritumoral AT, [^18^F]FDG PET imaging features of peritumoral AT could reflect this interaction and have a significant association with cancer progression in patients with gastric cancer. Recently, radiomic analysis, which extracts a large number of quantitative features from diagnostic imaging examinations through imaging processing methods, such as textural analysis, has been shown to provide deep and valuable insights into malignant diseases [19]. In previous studies with malignant diseases, first-order features based on standard uptake value (SUV) histograms, which measured diverse parameters related with SUV distribution, and second-order grey-level co-occurrence matrix (GLCM) features, which showed the SUV intensity level distribution in a neighborhood, have been generally used as radiomic features of PET images [17,20]. These first-order and second-order features have demonstrated superior values in detecting metastasis and predicting prognosis than conventional [^18^F]FDG PET/CT parameters such as the maximum SUV [17,21,22]. Likewise, textural features of peritumoral AT extracted from [^18^F]FDG PET images could aid in further understanding the significance of the imaging findings of peritumoral AT. However, to date, the prognostic value of SUV of visceral AT has been only evaluated in patients with gastric cancer, and there is no study that investigated the clinical significance of [^18^F]FDG PET textural features of peritumoral AT [23]. Furthermore, the relationship between [^18^F]FDG PET textural features and histopathological findings in peritumoral AT has not yet been reported.

In the present study, we extracted textural features of perigastric AT from staging [^18^F]FDG PET/CT images of retrospectively enrolled patients with gastric cancer and investigated whether these textural features had a significant relationship with the histopathological findings of perigastric AT and recurrence-free survival (RFS) after curative surgical resection.

## 2. Results

### 2.1. Patient Characteristics

The baseline characteristics of the 69 enrolled patients with gastric cancer are listed in Table 1. Of all patients, 9 patients (13.0%) had early gastric cancer (pT1 stage), and 60 patients (87.0%) had advanced gastric cancer (pT2–T4 stages). On [^18^F]FDG PET/CT, gastric cancer lesions in 52 patients (75.4%) showed an abnormally increased [^18^F]FDG uptake compared with the surrounding normal gastric wall uptake. The median follow-up duration was 46.8 months (range: 0.6–107.0 months). During the follow-up, 25 patients (36.2%) had events (recurrence or death).

### 2.2. Correlation Analysis between PET Textural Features and Histopathological Results

A comparative analysis of the maximum SUV of gastric cancer and textural features of perigastric AT according to the histopathological results of perigastric AT is shown in Table 2, Tables S1–S5. The results of the analysis revealed that CD163 cell infiltration grade was significantly correlated with the maximum SUV of the primary tumor and SUV mean, SUV histogram entropy, GLCM energy, and GLCM entropy of perigastric AT (*p* < 0.05). Moreover, interleukin-6 (IL-6) expression grade was significantly correlated with the mean and median SUVs of perigastric AT (*p* < 0.05). None of the PET imaging features showed a significant relationship with CD4 and CD8 cell infiltration grades and matrix metalloproteinase-11 (MMP-11) expression grade (*p* > 0.05). Post-hoc analysis showed that patients with grade 3 CD163 cell infiltration exhibited significantly higher values of the maximum SUV of the primary tumor and SUV mean, SUV histogram entropy, and GLCM entropy of perigastric AT and significantly lower values of GLCM energy of perigastric AT than those with grade 0 (*p* < 0.05; Table S3). Furthermore, patients with grade 3 IL-6 expression had significantly higher mean and median SUVs of perigastric AT than those with grade 0 expression (*p* < 0.05; Table S5).

### 2.3. Survival Analysis for RFS

The prognostic significance of PET imaging parameters for predicting RFS was assessed along with the clinicopathological factors. Of the PET textural features in perigastric AT, SUV mean, SUV median, SUV histogram energy, SUV histogram entropy, GLCM contrast, GLCM dissimilarity, GLCM energy, GLCM entropy, and GLCM homogeneity were significantly associated with RFS in the univariate analysis (*p* < 0.05; Table 3). In addition, the maximum SUV of the primary tumor, pT stage, pN stage, and TNM stage were significant predictors of RFS (*p* < 0.05).

For the textural features of perigastric AT that showed statistical significance in univariate analysis, the prognostic values for predicting RFS were further evaluated using multivariate analysis (Table 4). Because all the perigastric AT imaging features selected for multivariate analysis showed significant correlations with each other (*p* < 0.05), the prognostic significance of each perigastric AT imaging feature was assessed on separate models. Considering the numbers of variables as compared with the number of patients with recurrence, only age, sex, TNM stage, and the maximum SUV of primary tumor were additionally included in the multivariate analysis as covariates. Among the second-order GLCM PET features, GLCM dissimilarity, GLCM entropy, and GLCM homogeneity remained significant predictors of RFS in multivariate analysis (*p* < 0.05; Table 4). In contrast, none of the first-order PET features showed statistical significance (*p* > 0.05). An increase in GLCM dissimilarity and GLCM entropy was associated with an increased risk of recurrence, whereas an increase in GLCM homogeneity was associated with a decreased risk of recurrence.

**Figure 1:**
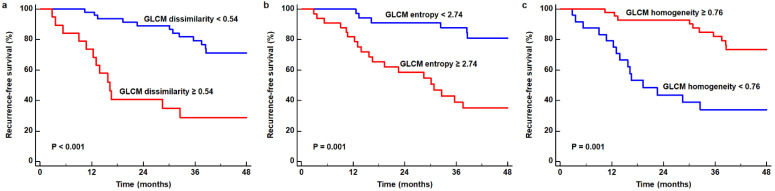
Cumulative recurrence-free survival curves based on the GLCM dissimilarity (**a**), GLCM entropy (**b**), and GLCM homogeneity (**c**) of perigastric AT.

In the Kaplan−Meier analysis, enrolled patients were classified into two groups according to the optimal cut-off values of GLCM dissimilarity (0.54), GLCM entropy (2.74), and GLCM homogeneity (0.76), as determined by the receiver operating characteristics (ROC) curve analysis. The results of the analysis demonstrated that patients with high values of GLCM dissimilarity and GLCM entropy had significantly worse 2-year RFS than those with low values (*p* < 0.001, 40.5% vs. 89.1% for GLCM dissimilarity; *p* = 0.001, 58.6% vs. 91.2% for GLCM entropy; Figure 1a,b). Patients with high GLCM homogeneity values showed a significantly better 2-year RFS than those with low values (*p* = 0.001, 92.9% vs. 43.6%; Figure 1c).

## 3. Discussion

Recently, ample evidence has been shown that the cross-talk between cancer cells and neighboring adipocytes significantly contribute to tumor growth and metastasis [6,7]. Upon interaction with cancer cells, adipocytes actively secrete proinflammatory and protumor adipokines, which induce inflammatory cell infiltration in the AT and destroy the extracellular matrix [6,8,9,24]. In terms of metabolic alterations, adipocytes lose their intracellular lipid content and provide exogenous fatty acids to cancer cells [7]. Since these morphological and functional modifications of adipocytes and inflammatory responses in the AT would bring qualitative changes in AT, a number of studies have tried to investigate whether the parameters of AT on diagnostic imaging examinations could represent these changes and could therefore be used as predictive factors for cancer progression [16,18,23,25]. On [^18^F]FDG PET/CT, the mean SUV of visceral AT was proposed to be an imaging parameter that could reflect these qualitative changes in AT [16,18,23,26,27]. In previous studies, the mean SUV of visceral AT demonstrated significant positive associations with tumor stage and survival after treatments in patients with various intra-abdominal malignancies including colorectal cancer, gastric cancer, and pancreatic cancer [16,23,26,27]. In addition to the mean SUV, we also measured the first-order and second-order textural features of perigastric AT and evaluated the relationship between these imaging features and histopathological findings, which could provide a relevant basis for using textural features of perigastric AT as imaging biomarkers. In the correlation analysis with histopathological findings, macrophage infiltration showed significant positive correlations with SUV mean, SUV histogram entropy, and GLCM entropy and significant negative correlations with GLCM energy. Furthermore, the mean and median SUVs showed significant positive correlations with IL-6 expression. Entropy measures the randomness of the intensity distribution in an image, and a high level of entropy represents randomly distributed intensities of voxels [17,28]. In contrast, energy measures the uniformity of the intensity distribution and is the opposite of entropy [28]. Therefore, the results of our study indicated that patients with increased macrophage infiltration and Il-6 expression in the perigastric AT had significantly higher values of [^18^F]FDG uptake and increased metabolic heterogeneity in the perigastric AT. 

Macrophages are the most abundant immune cells in the tumor microenvironment [29]. The majority of macrophages in peritumoral AT are M2 macrophages, which exhibit high levels of CD163 expression [8,29]. M2 macrophages are known to promote progression and metastasis of gastric cancer cells [29,30]. A recent study demonstrated a positive feedback loop between gastric cancer cells and M2 macrophages, in which gastric cancer cells induce M2-type polarization and M2 macrophages promote cancer progression and immune suppression in the tumor microenvironment [29]. IL-6 is a proinflammatory cytokine secreted by cancer cells, cancer stromal cells, and adipocytes as well as immune cells [24,31]. IL-6 secreted by dysfunctional adipocytes recruits macrophages in the AT, which leads to an inflammatory response in the AT, and IL-6 derived from tumor tissue plays an important role in the M2-subtype polarization of macrophages in peritumoral tissue [24,30]. M2 macrophages recruited to the tumor microenvironment can also release IL-6, which promotes programmed cell death-ligand 1 (PD-L1) expression and proliferation of gastric cancer cells [32]. Hence, macrophages and IL-6 are hypothesized to play crucial roles in the cross-talk between gastric cancer cells and the immune microenvironment, and they also showed significant prognostic value in patients with gastric cancer [33,34]. Considering the significant relationships of [^18^F]FDG PET/CT textural features with macrophage infiltration and IL-6 expression as shown in our study, [^18^F]FDG PET/CT textural features of perigastric AT might be potential imaging biomarkers for assessing immune microenvironment status in gastric cancer. Further immune profiling studies using multiplex immunohistochemistry analysis or flow cytometry analysis are needed to validate the use of textural features of perigastric AT as imaging biomarkers. Currently, M2 macrophages are potential therapeutic targets for treating gastric cancer, and several attempts have been made to limit macrophage recruitment or repolarize M2 macrophages in the tumor microenvironment [30,35]. The textural features of perigastric AT might be used to select optimal candidates and assess treatment effects in future clinical studies targeting M2 macrophages. 

Based on the results of the correlation analysis with histopathological findings, we further investigated the prognostic significance of textural features of perigastric AT for predicting RFS. Among the textural features that had a significant relationship with the histopathological findings, only GLCM entropy was significantly associated with RFS in multivariate analysis, showing worse survival in patients with high GLCM entropy. GLCM entropy is shown to have high robustness, irrespective of the iteration number, noise, image reconstruction algorithm, and matrix size of PET images; therefore, it is suggested as a suitable textural feature for use in multi-scanner studies [36]. Furthermore, in a previous study of breast cancer patients, GLCM entropy measured from peritumoral breast AT reflected the degree of the host response to metastatic lesion burden and showed a high diagnostic accuracy in predicting axillary lymph node metastasis [17]. Hence, GLCM entropy could be the most suitable imaging biomarker for representing the metabolic heterogeneity of peritumoral AT and could be preferentially recommended in future studies that investigate the clinical significance of peritumoral AT textural features. Along with GLCM entropy, GLCM dissimilarity and GLCM homogeneity were also significant predictors of RFS in multivariate analysis, although these two features had no significant relationship with the histopathological results. Dissimilarity-measured local contrasts and low dissimilarity values indicate that the intensities of neighboring voxels are very similar [28]. Homogeneity refers to the uniformity of a voxel pair, and a high value of homogeneity indicates that the image has many voxels with similar intensities or repetitive structures [28]. Since patients with high values of GLCM entropy and GLCM dissimilarity had a worse prognosis and patients with high values of GLCM homogeneity had a better prognosis, our results indicated that increased metabolic heterogeneity in the perigastric AT had a significant association with worse survival in patients with gastric cancer. In contrast, parameters regarding the degree of metabolism in perigastric AT, such as SUV mean, failed to show prognostic significance in multivariate survival analysis. On histopathological analysis, only the intensity of the inflammatory response in perigastric AT was assessed, and the distribution of the inflammatory response could not be evaluated [16]; this could be the reason for the insignificant relationship between histopathological results and GLCM dissimilarity and GLCM homogeneity. Therefore, a new histopathological analytical method that could estimate the distribution of immune responses in peritumoral AT might pave the way for a new perspective on the interactions between cancer and host cells. 

In addition to the textural features of perigastric AT, the maximum SUV of gastric cancer showed a significant positive association with macrophage infiltration and a borderline significant association with IL-6 expression in perigastric AT. The maximum SUV of primary tumor is known to be related to histopathologic types and biological characteristics of gastric cancer, and a significantly increased maximum SUV was shown in gastric cancers with positive PD-L1 expression when compared to those with negative expression [5,37,38]. The results of our study suggest that gastric cancers with aggressive biological features had a more enhanced immune response in perigastric AT. Furthermore, considering that M2 macrophages promote PD-L1 expression in gastric cancer cells through IL-6 secretion [32], our results suggest the possibility of a significant correlation between the maximum SUV and PD-L1 expression, which needs to be thoroughly assessed in future studies. 

The present study had several limitations. First, our study was retrospectively performed at a single medical center. Hence, there might be a certain risk of bias. Moreover, because this study enrolled only a small number of patients, other significant relationships between [^18^F]FDG PET/CT textural features and histopathological findings in peritumoral AT might be found in studies with a larger population. Second, although a large number of studies have reported the clinical implication of [^18^F]FDG PET/CT textural features, general applications of textural features in the real clinical world are still limited mainly due to the lack of standardization [39]. In previous studies, various factors including the voxel size, the image acquisition, and the reconstruction method, segmentation, and image analysis software can affect the reproducibility of textural features [39,40,41]. Third, a certain volume of perigastric AT is required to extract PET/CT textural features; therefore, the application of our imaging analytic method is limited to patients with extremely low volumes of perigastric AT. Fourth, although previous studies on breast and colorectal cancers also used a 1 cm distance to the tumor margin for measuring peritumoral AT imaging features, there is still no established methodology for defining peritumoral AT on [^18^F]FDG PET/CT images [16,17]. Finally, because up to 24% of patients with gastric cancer can have tumor deposits in the perigastric AT [42], the possible presence of tumor deposits might have affected the results of our study.

## 4. Materials and Methods

### 4.1. Patient Selection

We retrospectively reviewed the medical records and images of patients who had histopathological diagnosis of gastric cancer and underwent [^18^F]FDG PET/CT for staging work-up of gastric cancer between March 2012 and March 2020 at our medical center. A total of 69 patients who underwent curative surgical resection for gastric cancer were enrolled in our study. We excluded the following patients: (1) patients who showed distant metastasis on staging imaging examinations or peritoneal seeding metastases on surgical exploration, (2) patients who received any kind of neoadjuvant treatment before the surgery or received palliative curative surgical resection, (3) patients who had a previous history of another malignant disease or major abdominal surgery, (4) patients who showed an insufficient perigastric AT volume for calculating textural features, (5) patients who had inadequate surgical specimens for analyzing histopathology of perigastric AT, and (6) patients who were lost to follow-up without recurrence or death within 24 months after the surgery. 

All enrolled patients underwent blood tests, gastroduodenoscopy, contrast-enhanced abdominopelvic CT, and [^18^F]FDG PET/CT for staging work-up of gastric cancer. Based on the results of the staging examinations, curative subtotal or total gastrectomy with at least D1 lymphadenectomy was performed. The median interval between [^18^F]FDG PET/CT and surgery was four days (range: 1–28 days). Following curative surgical resection, adjuvant chemotherapy was recommended for patients with TNM stages II–III disease. After the treatment, a regular clinical follow-up was performed at intervals of 6–8 months during the first 3 years and 10–12 months thereafter, with routine examinations including blood tests, gastroduodenoscopy, and contrast-enhanced abdominopelvic CT.

### 4.2. [^18^F]FDG PET/CT and Image Analysis

All enrolled patients fasted for at least 6 h prior to the [^18^F]FDG PET/CT scan, and the blood glucose levels measured before the injection of [^18^F]FDG were <200 mg/dL. One hour after the intravenous administration of [^18^F]FDG (approximately 4.07 MBq per kg), [^18^F]FDG PET/CT scans were obtained using a dedicated scanner (Biograph mCT 128 scanner, Siemens Healthineers, Knoxville, TN, USA) from the skull base to the proximal thigh. Initially, a non-contrast-enhanced CT scan was performed for attenuation correction (100 mA and 120 kVp with an automated dose modulation, a slice thickness of 5 mm, and a slice increment of 2.5 mm). Afterwards, a PET scan was performed with a 1.5 min per bed position in the three-dimensional acquisition mode. An ordered subset expectation maximization reconstruction algorithm including time-of-flight information, point-spread-function based resolution recovery (TrueX), and attenuation correction was used for the PET image reconstruction on a 128 × 128 matrix (21 subsets and 2 iterations). 

**Figure 2:**
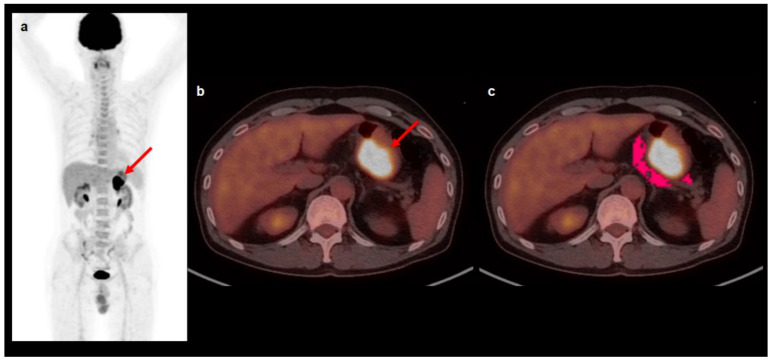
Maximal intensity projection image (**a**) and transaxial images (**b**,**c**) of [^18^F]FDG PET/CT illustrating VOI for measuring textural features of perigastric AT. A 59-year-old man underwent [^18^F]FDG PET/CT for staging work-up of gastric cancer in the stomach body. The gastric cancer lesion showed intensely increased [^18^F]FDG with the maximum SUV of 17.99 (arrows on (**a**,**b**)). A VOI that covers the area within a 1 cm distance to the margin of primary gastric cancer was manually drawn, and perigastric AT was defined as an area of CT-attenuation range between −190 HU and −30 HU within the VOI (red area in (**c**). The mean SUV, GLCM homogeneity, GLCM entropy, and GLCM dissimilarity of the perigastric AT were 1.05, 0.74, 3.01, and 0.49, respectively. The patient underwent total gastrectomy and was diagnosed with pT2N0 stage moderately differentiated tubular adenocarcinoma. The patient had cancer recurrence 23.9 months after the surgery.

Two nuclear medicine physicians separately reviewed the [^18^F]FDG PET/CT images and measured the imaging features of primary gastric cancer and perigastric AT using the LIFEx software (version 7.0.0; www.lifexsoft.org, accessed on 1 May 2022) [43]. During image analysis, the two reviewers were unaware of the clinical and histopathological results of the patients. First, the volume of interest (VOI) was manually constructed over the primary gastric cancer lesion, and then, the highest value of [^18^F]FDG uptake in the VOI (the maximum SUV of the primary tumor) was measured. In patients with gastric cancer lesions that could not be differentiated from the surrounding normal gastric wall uptake, the VOI of the primary tumor lesion was drawn in accordance with the tumor location observed on endoscopy and contrast-enhanced CT images. Subsequently, a VOI that covered the area within a 1 cm distance to the margin of the primary gastric cancer lesion was manually drawn. Within the VOI, an area of CT-attenuation ranging from −190 Hounsfield unit (HU) to −30 HU was selected and defined as perigastric AT (Figure 2) [16,17,44]. Before extracting imaging features, all perigastric AT areas were manually scrutinized to prevent spillover [^18^F]FDG activity of primary cancer lesions in the areas. From the perigastric AT area, 13 textural features of PET images including seven first-order features and six second-order features derived from the GLCM were extracted for each patient. GLCM considers the spatial distribution of intensity levels in pairs of voxels [28]. Seven first-order features comprised of the mean (SUV mean), standard deviation (SUV std), and median (SUV median) SUV of perigastric AT and kurtosis (SUV histogram kurtosis), skewness (SUV histogram skewness), energy (SUV histogram energy), and entropy (SUV histogram entropy) based on the SUV histogram. The GLCM was calculated from 13 directions in three-dimensional space, and six GLCM features (contrast, correlation, dissimilarity, energy, entropy, and homogeneity) were extracted from the PET images.

### 4.3. Histopathological Analysis

**Figure 3:**
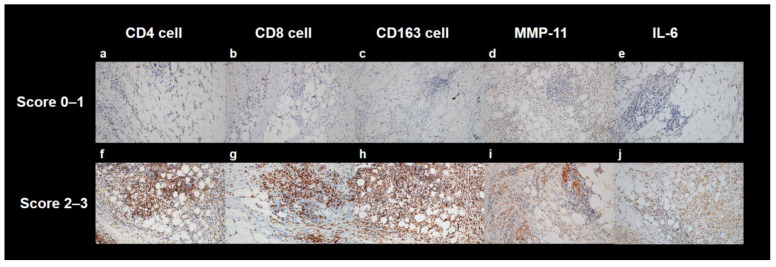
Representative images of immunohistochemical analyses of the perigastric AT. Immunohistochemical staining of CD4 (**a**,**f**), CD8 (**b**,**g**), CD163 (**c**,**h**), MMP-11 (**d**,**i**), and IL-6 (**e**,**j**). Examples of a score range of 0–1 are shown in (**a**–**e**), and examples of a score range of 2–3 area shown in (**f**–**j**). The magnifications of all images were 200×.

The surgical specimens of the patients were retrospectively reviewed by two pathologists. Gastric cancer lesions were histopathologically classified into five subtypes: papillary adenocarcinoma, well-to-moderately differentiated tubular adenocarcinoma, poorly differentiated adenocarcinoma, mucinous carcinoma, and signet ring cell carcinoma [23]. The microscopic growth patterns of gastric cancers were categorized into two subtypes based on the Lauren classification: intestinal and non-intestinal types. Non-intestinal types included diffuse, mixed, and non-classifiable types [23]. The histopathological T and N stages of gastric cancers were assessed according to the eighth edition of the American Joint Committee on Cancer staging guidelines. To evaluate inflammatory response of perigastric AT, infiltrations of immune cells, such as T cells and macrophages, and expressions of protein and cytokine related to inflammatory response, such as MMP-11 and IL-6, were assessed with immunohistochemical analysis. For immunohistochemical analysis of perigastric AT, hematoxylin and eosin-stained slides were made from formalin-fixed, paraffin-embedded tissue blocks, and they were reviewed under a light microscope to select perigastric AT (immediately close to the cancer cells). The corresponding areas of each paraffin block were cored twice with a 2 mm-diameter cylinder and assembled into a recipient paraffin block using a tissue microarray (TMA) instrument (Unitma, Seoul, Korea). We performed immunohistochemical staining of individual 4-μm thick slide sections derived from TMA blocks using the Ventana Benchmark XT automated staining system (Ventana Medical Systems, Tucson, AZ, USA) according to the established protocol of the manufacturer. For immunohistochemical analysis, we used the following anti-bodies: monoclonal rabbit anti-human CD4 (clone SP35, Catalog no. 7904423, Ventana Medical System), monoclonal mouse anti-human CD8 (clone C8/144B, Catalog no. IR623, Dako, Carpinteria, CA, USA), monoclonal mouse anti-human CD163 (clone OTI2G12, Catalog no. ab156769, Abcam, Cambridge, UK), monoclonal rabbit anti-human MMP-11 (clone SN74-08, Catalog no. NBP2-67670, Novus Biologicals, Centennial, CO, USA), and polyclonal rabbit anti-human IL-6 (Catalog no. ab6672, Abcam). Three representative areas under a high-power optical microscope (magnification: 400×) were selected from each core of TMA. The numbers of CD4+, CD8+, and CD163+ infiltrating cells were rated as follows: 0, (absent); 1, (1–25 cells); 2, (26–50 cells); and 3, (>50 cells) (Figure 3). To score the expression of MMP-11 and IL-6, the intensity score was based off this system: 0, negative; 1, focal light brown (weak); 2, light brown (moderate) and 3, brown (marked) (Figure 3).

### 4.4. Statistical Analysis

**Figure 4:**
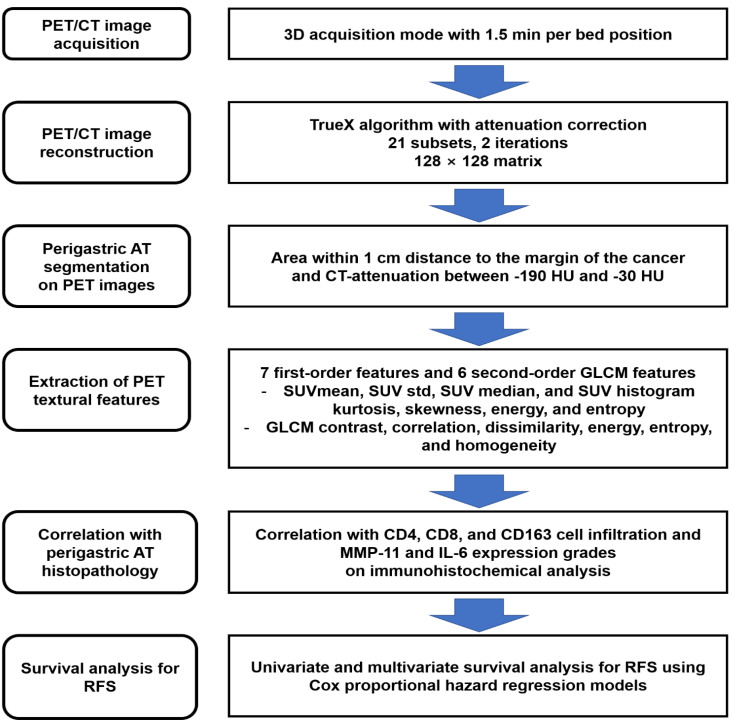
Schematic flowchart of the overall workflow.

The overall workflow in the present study is presented in Figure 4. The Kruskal−Wallis test with post-hoc analysis using Dunn’s test was performed to evaluate the relationship between PET imaging parameters and the histopathological results of perigastric AT. The prognostic value of PET textural features of perigastric AT for RFS was assessed using univariate and multivariate Cox proportional hazard regression models. Survival time was defined as the time from the day of surgical resection to the day of cancer recurrence (or death). Patients with no events were censored on the day of the last follow-up visit. PET textural features of perigastric AT that showed statistical significance in predicting RFS on univariate analysis were selected for multivariate survival analysis. In multivariate survival analysis, the prognostic significance of each perigastric AT imaging feature was assessed by adding age, sex, TNM stage, and the maximum SUV of primary tumor as covariates for the analysis. PET imaging features that were significantly correlated were evaluated using a separate model. For the Kaplan−Meier analysis, the specific cut-off values of PET textural features of perigastric AT were selected using ROC curve analysis. The optimal cut-off values were determined by using the Youden index. Based on the cut-off values, the patients were dichotomized, and the cumulative RFS curve for each group was estimated using the Kaplan−Meier method. Statistical analyses were performed using MedCalc Statistical Software version 20.110 (MedCalc Software Ltd., Ostend, Belgium). Statistical significance was set at *p* < 0.05.

## 5. Conclusions

In conclusion, textural features of perigastric AT on [^18^F]FDG PET/CT were significantly associated with macrophage infiltration and IL-6 expression in perigastric AT; additionally, these were significant predictors of RFS in patients with gastric cancer. Increased metabolic heterogeneity in perigastric AT was associated with a severe inflammatory response in the perigastric AT and an increased risk of recurrence after curative surgery. Our results suggested that textural features of perigastric AT on [^18^F]FDG PET/CT might be potential imaging biomarkers for evaluating status of perigastric AT and predicting prognosis of patients with gastric cancer; however, further validations of the results in future prospective studies are necessary.

## Figures and Tables

**Table 1:** Characteristics of the 69 enrolled patients with gastric cancer.

Variables		Number of Patients (%)
Age (years)		60 (34–80) *
Sex	Men	39 (56.5%)
	Women	30 (43.5%)
Tumor location	Upper	7 (10.1%)
	Middle	28 (40.6%)
	Lower	34 (49.3%)
Histopathological classification	PAC/TAC	43 (62.3%)
	PDAC	16 (23.2%)
	Mucinous carcinoma/SRC	10 (14.5%)
Lauren classification	Intestinal	30 (43.5%)
	Non-intestinal	39 (56.5%)
pT stage	T1 stage	9 (13.0%)
	T2 stage	17 (24.6%)
	T3 stage	23 (33.3%)
	T4 stage	20 (29.0%)
pN stage	N0 stage	29 (42.0%)
	N1−N3 stages	40 (58.0%)
TNM stage	Stage I	18 (26.1%)
	Stage II	19 (27.5%)
	Stage III	32 (46.4%)
Adjuvant treatment	Yes	41 (59.4%)
	No	28 (40.6%)
CD4 cell infiltration	Grade 0	17 (24.6%)
	Grade 1	17 (24.6%)
	Grade 2	23 (33.3%)
	Grade 3	12 (17.4%)
CD8 cell infiltration	Grade 0	16 (23.2%)
	Grade 1	17 (24.6%)
	Grade 2	19 (27.5%)
	Grade 3	17 (24.6%)
CD163 cell infiltration	Grade 0	10 (14.5%)
	Grade 1	20 (29.0%)
	Grade 2	23 (33.3%)
	Grade 3	16 (23.2%)
MMP-11 expression	Grade 0	14 (20.3%)
	Grade 1	22 (31.9%)
	Grade 2	23 (33.3%)
	Grade 3	10 (14.5%)
IL-6 expression	Grade 0	28 (40.6%)
	Grade 1	24 (34.8%)
	Grade 2	12 (17.4%)
	Grade 3	5 (7.2%)

* Expressed as a median (range). IL-6, interleukin-6; MMP-11, matrix metalloproteinase-11; PAC, papillary adenocarcinoma; PDAC, poorly differentiated adenocarcinoma; SRC, signet ring cell carcinoma; TAC, well-to-moderately differentiated tubular adenocarcinoma.

**Table 2:** Statistical significance of the Kruskal−Wallis test for comparing the maximum SUV of primary tumor and perigastric AT imaging features according to the histopathological results of perigastric AT.

Variables	CD4 Cell Infiltration	CD8 Cell Infiltration	CD163 Cell Infiltration	MMP-11 Expression	IL-6 Expression
Maximum SUV of primary tumor	0.078	0.149	0.006	0.458	0.094
First-order PET features of perigastric AT					
SUV mean	0.163	0.072	0.037	0.099	0.042
SUV std	0.469	0.949	0.402	0.583	0.343
SUV median	0.189	0.062	0.095	0.122	0.025
SUV histogram kurtosis	0.699	0.911	0.330	0.714	0.869
SUV histogram skewness	0.470	0.387	0.496	0.226	0.170
SUV histogram energy	0.191	0.661	0.095	0.596	0.261
SUV histogram entropy	0.320	0.537	0.030	0.392	0.493
Second-order PET features of perigastric AT					
GLCM contrast	0.700	0.556	0.216	0.687	0.638
GLCM correlation	0.386	0.072	0.356	0.469	0.292
GLCM dissimilarity	0.648	0.622	0.097	0.721	0.830
GLCM energy	0.122	0.145	0.023	0.752	0.066
GLCM entropy	0.106	0.192	0.035	0.296	0.097
GLCM homogeneity	0.612	0.325	0.115	0.721	0.927

AT, adipose tissue; GLCM, grey-level co-occurrence matrix; IL-6, interleukin-6; MMP-11, matrix metalloproteinase-11; PET, positron emission tomography; std, standard deviation; SUV, standardized uptake value.

**Table 3:** Results of univariate analysis for recurrence-free survival.

Variables		*p*-Value	Hazard Ratio (95% CI)
Age (for 1-year increase)		0.997	1.00 (0.97–1.04)
Sex (women vs. men)		0.295	1.63 (0.65–4.10)
Histopathological classification (PAC/TAC vs.)	PDAC	0.234	1.71 (0.71–4.13)
	Mucinous/SRC	0.196	1.42 (0.86–4.38)
Lauren classification (intestinal vs. non-intestinal)		0.553	1.27 (0.57–2.84)
pT stage (T1–T2 vs. T3–T4)		0.003	20.95 (2.83–155.14)
pN stage (N0 vs. N1–3)		<0.001	12.36 (2.90–52.61)
TNM stage (stages I−II vs. stage III)		<0.001	14.88 (4.41–50.20)
Maximum SUV of primary tumor (for 1.0 increase)		0.001	1.10 (1.04–1.16)
First-order PET features of perigastric AT (for a 0.10 increase)	SUV mean	<0.001	1.27 (1.12–1.46)
	SUV std	0.464	1.17 (0.77–1.75)
	SUV median	0.001	1.29 (1.13–1.47)
	SUV histogram kurtosis	0.785	1.00 (0.98–1.03)
	SUV histogram skewness	0.157	0.94 (0.86–1.02)
	SUV histogram energy	0.017	0.61 (0.41–0.91)
	SUV histogram entropy	0.019	1.11 (1.02–1.21)
Second-order PET features of perigastric AT (for a 0.10 increase)	GLCM contrast	0.004	1.13 (1.04–1.23)
	GLCM correlation	0.087	0.82 (0.65–1.03)
	GLCM dissimilarity	0.007	1.36 (1.14–1.62)
	GLCM energy	0.022	0.58 (0.36–0.92)
	GLCM entropy	<0.001	1.12 (1.05–1.20)
	GLCM homogeneity	0.003	0.40 (0.24–0.65)

AT, adipose tissue; CI, confidence interval; GLCM, grey-level co-occurrence matrix; PAC, papillary adenocarcinoma; PDAC, poorly differentiated adenocarcinoma; PET, positron emission tomography; SRC, signet ring cell carcinoma; std, standard deviation; SUV, standardized uptake value; TAC, well-to-moderately differentiated tubular adenocarcinoma.

**Table 4:** Results of multivariate analysis of PET textural features for recurrence-free survival after adjustment for age, sex, TNM stage, and the maximum SUV of primary tumor.

Variables		*p*-Value	Hazard Ratio (95% CI)
First-order PET features of perigastric AT (for a 0.10 increase)	SUV mean	0.147	
	SUV median	0.090	
	SUV histogram energy	0.222	
	SUV histogram entropy	0.409	
Second-order PET features of perigastric AT (for 0.10 increase)	GLCM contrast	0.464	
	GLCM dissimilarity	0.013	1.31 (1.06–1.62)
	GLCM energy	0.099	
	GLCM entropy	0.019	1.07 (1.02–1.15)
	GLCM homogeneity	0.012	0.42 (0.21–0.82)

AT, adipose tissue; CI, confidence interval; GLCM, grey-level co-occurrence matrix; PET, positron emission tomography; SUV, standardized uptake value.

## Data Availability

The datasets generated during and/or analyzed during the current study are available from the corresponding authors upon reasonable request.

## Supplementary Materials

The following supporting information can be downloaded at: https://www.mdpi.com/article/10.3390/ijms231911985/s1.
