# Supplementary material for: Predicting the Recurrence of Gastric Cancer Using the Textural Features of Perigastric Adipose Tissue on [18F]FDG PET/CT
Source: Int J Mol Sci. 2022 Oct 9;23(19):11985. doi: 10.3390/ijms231911985 (PMC9569486; doi:10.3390/ijms231911985)
Supplement: Supplementary file 1 [file ijms-23-11985-s001.zip › ijms-1946244-supplementary.pdf]

Article title: Predicting the recurrence of gastric cancer using the textural features of perigastric adipose tissue on [^{18}F]FDG PET/CT

Author names: Hyein Ahn, Geum Jong Song, Si-Hyong Jang, Myoung Won Son, Hyun Ju Lee, Moon-Soo Lee, Ji-Hye Lee, Mee-Hye Oh, Geum Cheol Jeong, Jong Hyuk Yun, Sang Mi Lee, Jeong Won Lee

Corresponding author: Sang Mi Lee, Department of Nuclear Medicine, Soonchunhyang University Cheonan Hospital

Co-corresponding author: Jeong Won Lee, Department of Nuclear Medicine, Catholic Kwandong University College of Medicine, International St. Mary's Hospital

Table S1. Comparisons of maximum standardized uptake value (SUV) of gastric cancer lesions and textural features of perigastric adipose tissue according to CD4 cell infiltration grades in perigastric adipose tissue.

Variables	Grade 0 (*n* = 17)	Grade 1 (*n* = 17)	Grade 2 (*n* = 23)	Grade 3 (*n* = 12)	P-value
Maximum SUV of primary tumor	3.20 (2.65–6.68)	3.88 (3.00–5.79)	4.90 (3.31–8.23)	6.36 (4.60–11.13)	0.078
First-order PET features of perigastric AT					
SUV mean	0.80 (0.65–1.09)	1.02 (0.77–1.11)	1.03 (0.90–1.23)	1.01 (0.91–1.33)	0.163
SUV std	0.25 (0.17–0.29)	0.22 (0.19–0.30)	0.26 (0.23–0.32)	0.25 (0.21–0.28)	0.469
SUV median	0.84 (0.56–0.99)	1.01 (0.74–1.13)	0.97 (0.87–1.29)	0.96 (0.86–1.33)	0.189
SUV histogram kurtosis	3.20 (2.82–3.62)	3.06 (2.78–4.46)	2.89 (2.50–3.90)	3.64 (2.49–5.28)	0.699
SUV histogram skewness	0.76 (0.63–0.95)	0.73 (0.36–1.25)	0.53 (0.28–0.97)	0.66 (0.50–1.12)	0.470
SUV histogram energy	0.35 (0.31–0.60)	0.36 (0.33–0.45)	0.33 (0.27–0.38)	0.39 (0.31–0.46)	0.191
SUV histogram entropy	1.73 (0.95–1.90)	1.61 (1.45–1.93)	1.88 (1.61–2.17)	1.63 (1.39–1.89)	0.320
Second-order PET features of perigastric AT					
GLCM contrast	0.50 (0.25–0.72)	0.43 (0.37–0.67)	0.55 (0.42–0.82)	0.46 (0.33–0.65)	0.700
GLCM correlation	0.58 (0.52–0.69)	0.59 (0.51–0.69)	0.56 (0.37–0.68)	0.54 (0.44–0.59)	0.386
GLCM dissimilarity	0.46 (0.25–0.57)	0.39 (0.35–0.55)	0.48 (0.39–0.64)	0.38 (0.32–0.54)	0.648
GLCM energy	0.23 (0.19–0.46)	0.23 (0.18–0.26)	0.18 (0.14–0.24)	0.26 (0.17–0.32)	0.122
GLCM entropy	2.68 (1.75–2.88)	2.66 (2.32–3.17)	2.92 (2.52–3.29)	2.31 (2.15–2.83)	0.106
GLCM homogeneity	0.78 (0.73–0.88)	0.81 (0.74–0.83)	0.78 (0.71–0.81)	0.82 (0.75–0.85)	0.612

Expressed in median (25th percentile–75th percentile)

AT, adipose tissue; GLCM, grey-level co-occurrence matrix; PET, positron emission tomography; std, standard deviation

Table S2. Comparisons of maximum standardized uptake value (SUV) of gastric cancer lesions and textural features of perigastric adipose tissue according to CD8 cell infiltration grades in perigastric adipose tissue.

Variables	Grade 0 (*n* = 16)	Grade 1 (*n* = 17)	Grade 2 (*n* = 19)	Grade 3 (*n* = 17)	P-value
Maximum SUV of primary tumor	3.80 (2.52–6.21)	3.39 (2.99–5.51)	5.09 (3.23–8.19)	4.99 (4.06–10.49)	0.149
First-order PET features of perigastric AT					
SUV mean	0.80 (0.67–1.04)	1.02 (0.77–1.09)	1.05 (1.01–1.40)	0.99 (0.88–1.21)	0.072
SUV std	0.25 (0.18–0.29)	0.25 (0.21–0.31)	0.26 (0.20–0.36)	0.27 (0.22–0.28)	0.949
SUV median	0.87 (0.61–0.98)	0.93 (0.71–1.11)	1.03 (0.95–1.39)	0.94 (0.82–1.22)	0.062
SUV histogram kurtosis	3.10 (2.81–3.79)	2.97 (2.46–4.12)	3.20 (2.52–4.05)	3.49 (2.47–5.02)	0.911
SUV histogram skewness	0.76 (0.54–0.94)	0.75 (0.38–1.25)	0.54 (0.14–0.85)	0.63 (0.36–1.08)	0.387
SUV histogram energy	0.38 (0.32–0.53)	0.35 (0.31–0.39)	0.36 (0.26–0.42)	0.33 (0.30–0.43)	0.661
SUV histogram entropy	1.62 (1.15–2.54)	1.79 (1.55–1.90)	1.70 (1.48–2.17)	1.88 (1.48–2.09)	0.537
Second-order PET features of perigastric AT					
GLCM contrast	0.46 (0.30–0.71)	0.50 (0.36–0.68)	0.49 (0.39–0.63)	0.60 (0.40–0.96)	0.556
GLCM correlation	0.60 (0.49–0.69)	0.58 (0.52–0.69)	0.57 (0.53–0.69)	0.46 (0.36–0.57)	0.072
GLCM dissimilarity	0.40 (0.29–0.54)	0.41 (0.35–0.57)	0.45 (0.36–0.53)	0.52 (0.33–0.67)	0.622
GLCM energy	0.27 (0.20–0.41)	0.23 (0.18–0.25)	0.19 (0.14–0.27)	0.18 (0.15–0.34)	0.145
GLCM entropy	2.34 (2.12–2.79)	2.74 (2.37–3.04)	2.85 (2.33–3.49)	2.83 (2.06–3.00)	0.192
GLCM homogeneity	0.81 (0.76–0.85)	0.80 (0.73–0.83)	0.78 (0.75–0.83)	0.76 (0.71–0.84)	0.325

Expressed in median (25th percentile–75th percentile)

AT, adipose tissue; GLCM, grey-level co-occurrence matrix; PET, positron emission tomography; std, standard deviation

Table S3. Comparisons of maximum standardized uptake value (SUV) of gastric cancer lesions and textural features of perigastric adipose tissue according to CD163 cell infiltration grades in perigastric adipose tissue.

Variables	Grade 0 (*n* = 10)	Grade 1 (*n* = 20)	Grade 2 (*n* = 23)	Grade 3 (*n* = 16)	P-value
Maximum SUV of primary tumor	2.62 (1.81–3.43)	4.14 (3.35–6.83)	4.64 (3.21–7.40)	6.80 (5.01–11.49)	**0.006***
First-order PET features of perigastric AT					
SUV mean	0.72 (0.59–1.01)	0.98 (0.76–1.34)	1.00 (0.84–1.18)	1.06 (1.02–1.28)	**0.037***
SUV std	0.19 (0.11–0.31)	0.25 (0.22–0.30)	0.26 (0.19–0.31)	0.26 (0.23–0.30)	0.402
SUV median	0.78 (0.56–0.93)	0.94 (0.71–1.23)	0.96 (0.83–1.46)	1.07 (0.92–1.32)	0.095
SUV histogram kurtosis	3.33 (2.36–4.12)	3.25 (2.81–4.28)	2.78 (2.36–3.49)	3.64 (2.63–4.33)	0.330
SUV histogram skewness	0.86 (0.54–1.00)	0.75 (0.58–0.93)	0.53 (0.23–0.78)	0.66 (0.39–1.23)	0.496
SUV histogram energy	0.55 (0.34–0.62)	0.35 (0.31–0.40)	0.34 (0.29–0.46)	0.34 (0.30–0.38)	0.095
SUV histogram entropy	1.06 (0.68–1.77)	1.76 (1.54–1.91)	1.75 (1.44–1.90)	1.88 (1.63–2.29)	**0.030***
Second-order PET features of perigastric AT					
GLCM contrast	0.35 (0.21–0.50)	0.50 (0.38–0.72)	0.55 (0.37–0.76)	0.54 (0.42–0.77)	0.216
GLCM correlation	0.60 (0.55–0.73)	0.58 (0.37–0.68)	0.55 (0.45–0.61)	0.56 (0.49–0.68)	0.356
GLCM dissimilarity	0.31 (0.21–0.46)	0.41 (0.35–0.60)	0.50 (0.37–0.60)	0.47 (0.38–0.58)	0.097
GLCM energy	0.33 (0.25–0.55)	0.23 (0.18–0.27)	0.18 (0.16–0.29)	0.19 (0.14–0.28)	**0.023†**
GLCM entropy	2.03 (1.49–2.66)	2.65 (2.36–2.91)	2.85 (2.23–3.19)	2.84 (2.24–3.22)	**0.035‡**
GLCM homogeneity	0.85 (0.78–0.90)	0.81 (0.72–0.83)	0.76 (0.73–0.83)	0.75 (0.71–0.82)	0.115

Expressed in median (25th percentile–75th percentile)

AT, adipose tissue; GLCM, grey-level co-occurrence matrix; PET, positron emission tomography; std, standard deviation

*On post-hoc analysis, patients with grade 3 had significantly higher values of parameters than those with grade 0 ($p<0.05$)

†On post-hoc analysis, patients with grade 2 and 3 had significantly lower values of parameters than those with grade 0 ($p<0.05$)

‡On post-hoc analysis, patients with grade 2 and 3 had significantly higher values of parameters than those with grade 0 ($p<0.05$)

Table S4. Comparisons of maximum standardized uptake value (SUV) of gastric cancer lesions and textural features of perigastric adipose tissue according to matrix metalloproteinase-11 expression grades in perigastric adipose tissue.

Variables	Grade 0 (*n* = 14)	Grade 1 (*n* = 22)	Grade 2 (*n* = 23)	Grade 3 (*n* = 10)	P-value
Maximum SUV of primary tumor	3.78 (2.46–4.90)	4.44 (3.23–0.12)	4.89 (3.36–7.43)	5.22 (2.83–10.10)	0.458
First-order PET features of perigastric AT					
SUV mean	0.91 (0.68–1.01)	1.03 (0.92–1.18)	0.90 (0.73–1.31)	1.08 (1.02–1.30)	0.099
SUV std	0.26 (2.22–0.31)	0.25 (0.18–0.30)	0.23 (0.19–0.28)	0.29 (0.23–0.36)	0.583
SUV median	0.86 (0.64–0.95)	0.98 (0.90–1.15)	0.91 (0.69–1.34)	1.09 (0.93–1.21)	0.122
SUV histogram kurtosis	3.30 (2.82–4.12)	2.99 (2.40–4.55)	3.07 (2.60–4.64)	3.17 (2.22–3.64)	0.714
SUV histogram skewness	0.91 (0.70–1.00)	0.63 (0.37–0.94)	0.72 (0.36–1.13)	0.48 (0.10–0.79)	0.226
SUV histogram energy	0.35 (0.32–0.40)	0.36 (0.30–0.49)	0.37 (0.30–0.46)	0.31 (0.29–0.36)	0.596
SUV histogram entropy	1.74 (1.53–1.84)	1.73 (1.43–1.91)	1.70 (1.39–2.05)	1.97 (1.70–2.29)	0.392
Second-order PET features of perigastric AT					
GLCM contrast	0.53 (0.31–0.72)	0.58 (0.37–0.80)	0.46 (0.38–0.55)	0.60 (0.40–0.93)	0.687
GLCM correlation	0.57 (0.53–0.64)	0.56 (0.42–0.63)	0.54 (0.45–0.68)	0.60 (0.58–0.64)	0.469
GLCM dissimilarity	0.48 (0.30–0.54)	0.50 (0.34–0.63)	0.41 (0.34–0.49)	0.49 (0.39–0.61)	0.721
GLCM energy	0.24 (0.18–0.29)	0.21 (0.14–0.34)	0.23 (0.18–0.33)	0.18 (0.15–0.23)	0.752
GLCM entropy	2.52 (2.30–3.13)	2.83 (2.17–3.20)	2.62 (2.14–2.92)	3.05 (2.63–3.23)	0.296
GLCM homogeneity	0.77 (0.75–0.85)	0.76 (0.71–0.84)	0.81 (0.76–0.84)	0.77 (0.73–0.81)	0.721

Expressed in median (25th percentile–75th percentile)

AT, adipose tissue; GLCM, grey-level co-occurrence matrix; PET, positron emission tomography; std, standard deviation

Table S5. Comparisons of maximum standardized uptake value (SUV) of gastric cancer lesions and textural features of perigastric adipose tissue according to interleukin-6 expression grades in perigastric adipose tissue.

Variables	Grade 0 (*n* = 28)	Grade 1 (*n* = 24)	Grade 2 (*n* = 12)	Grade 3 (*n* = 5)	P-value
Maximum SUV of primary tumor	3.92 (2.78–6.15)	4.95 (3.42–7.51)	4.14 (2.62–7.55)	10.66 (4.99–12.62)	0.094
First-order PET features of perigastric AT					
SUV mean	0.96 (0.69–1.08)	1.01 (0.84–1.16)	1.02 (0.90–1.26)	1.31 (1.22–1.50)	**0.042***
SUV std	0.25 (0.20–0.31)	0.26 (0.20–0.30)	0.22 (0.18–0.28)	0.31 (0.25–0.37)	0.343
SUV median	0.93 (0.67–1.06)	0.94 (0.80–1.12)	1.01 (0.91–1.26)	1.34 (1.25–1.48)	**0.025†**
SUV histogram kurtosis	3.19 (2.49–4.77)	3.12 (2.69–4.31)	2.89 (2.45–3.71)	3.37 (2.70–3.91)	0.869
SUV histogram skewness	0.76 (0.49–1.11)	0.72 (0.52–1.12)	0.43 (0.14–0.76)	0.51 (0.27–0.79)	0.170
SUV histogram energy	0.36 (0.30–0.51)	0.35 (0.32–0.41)	0.38 (0.30–0.45)	0.29 (0.23–0.33)	0.261
SUV histogram entropy	1.74 (1.27–1.90)	1.73 (1.49–1.91)	1.60 (1.42–1.99)	2.06 (1.79–2.30)	0.493
Second-order PET features of perigastric AT					
GLCM contrast	0.52 (0.32–0.72)	0.47 (0.38–0.70)	0.45 (0.36–0.56)	0.63 (0.49–0.85)	0.638
GLCM correlation	0.57 (0.48–0.63)	0.57 (0.51–0.68)	0.51 (0.39–0.60)	0.68 (0.58–0.74)	0.292
GLCM dissimilarity	0.47 (0.31–0.58)	0.41 (0.36–0.56)	0.43 (0.33–0.50)	0.48 (0.42–0.65)	0.830
GLCM energy	0.25 (0.18–0.35)	0.23 (0.19–0.29)	0.20 (0.15–0.28)	0.13 (0.11–0.16)	0.066
GLCM entropy	2.48 (2.15–2.94)	2.68 (2.20–2.97)	2.75 (2.19–3.14)	3.14 (2.84–3.47)	0.097
GLCM homogeneity	0.78 (0.73–0.85)	0.81 (0.74–0.83)	0.76 (0.76–0.84)	0.78 (0.71–0.80)	0.927

Expressed in median (25th percentile–75th percentile)

AT, adipose tissue; GLCM, grey-level co-occurrence matrix; PET, positron emission tomography; std, standard deviation

*On post-hoc analysis, patients with grade 3 had significantly higher values of parameters than those with grade 0 ($p<0.05$)

† On post-hoc analysis, patients with grade 3 had significantly higher values of parameters than those with grade 0 and 1 ($p<0.05$)
